# Time-dependent postural control adaptations following a neuromuscular warm-up in female handball players: a randomized controlled trial

**DOI:** 10.1186/s13102-016-0058-5

**Published:** 2016-10-13

**Authors:** Simon Steib, Peter Zahn, Christine zu Eulenburg, Klaus Pfeifer, Astrid Zech

**Affiliations:** 1 Institute of Sport Science and Sport, Division of Exercise and Health, Friedrich-Alexander University Erlangen-Nürnberg, Erlangen, Germany; 2 University Medical Center Groningen, Faculty of Medical Sciences, University of Groningen, Groningen, Netherlands; 3 Department of Sport Science, Friedrich Schiller University Jena, Jena, Germany

**Keywords:** Balance, Postural control, Sensorimotor training, Neuromuscular training, Injury prevention

## Abstract

**Background:**

Female handball athletes are at a particular risk of sustaining lower extremity injuries. The study examines time-dependent adaptations of static and dynamic balance as potential injury risk factors to a specific warm-up program focusing on neuromuscular control.

**Methods:**

Fourty one (24.0 ± 5.9 years) female handball athletes were randomized to an intervention or control group. The intervention group implemented a 15-min specific neuromuscular warm-up program, three times per week for eleven weeks, whereas the control group continued with their regular warm-up. Balance was assessed at five time points. Measures included the star excursion balance test (SEBT), and center of pressure (COP) sway velocity during single-leg standing.

**Results:**

No baseline differences existed between groups in demographic data. Adherence to neuromuscular warm-up was 88.7 %. Mean COP sway velocity decreased significantly over time in the intervention group (−14.4 %; *p* < .001), but not in the control group (−6.2 %; *p* = 0.056). However, these effects did not differ significantly between groups (*p* = .098). Mean changes over time in the SEBT score were significantly greater (*p* = .014) in the intervention group (+5.48) compared to the control group (+3.45). Paired t-tests revealed that the first significant balance improvements were observed after 6 weeks of training.

**Conclusions:**

A neuromuscular warm-up positively influences balance variables associated with an increased risk of lower extremity injuries in female handball athletes. The course of adaptations suggests that a training volume of 15 min, three times weekly over at least six weeks produces measurable changes.

**Trial registration:**

Retrospectively registered on 4th October 2016. Registry: clinicaltrials.gov. Trial number: NCT02925377.

**Electronic supplementary material:**

The online version of this article (doi:10.1186/s13102-016-0058-5) contains supplementary material, which is available to authorized users.

## Background

Team ball sports athletes, such as handball players, are at a high risk of injury [1]. Female players are at a particular risk of sustaining ligamentous injuries to the knee and ankle joint. Long-term studies suggest a 3- to 5-times increased risk of sustaining ACL injuries in team ball sports [2, 3]. Deficits in static and dynamic balance, indicating the athletes’ ability to control their center of mass over the base of support during standing (static) or moving (dynamic), have been discussed as important intrinsic risk factors of sustaining lower extremity injuries [4, 5].

Neuromuscular training programs are increasingly implemented in professional and amateur team sport athletes [6–8]. They are widely accepted to be effective for the prevention of lower extremity injuries [9–12]. However, the underlying mechanisms are not yet fully understood. Preventive effects are generally attributed to a modification of various intrinsic injury risk factors, including static and dynamic balance [13–16].

The individual components of preventive neuromuscular training interventions, as well as their dosage vary substantially between trials [11, 17, 18]. The regular incorporation of neuromuscular training components into the athletes’ warm-up routine as a frequent and economic alternative to high volume programs has become increasingly popular and has demonstrated promising effects [19, 20]. However, to date only little evidence exists regarding the dimensions, time course, and magnitude of adaptations to neuromuscular warm-up programs. Preliminary findings suggest that adaptations of balance appear to vary depending on content and dosage of interventions, as well as the population trained [13, 14, 16, 21]. Thus, the identification of causal relations between neuromuscular training specifications and adaptations in individual injury risk factors is a vital step in promoting a more differentiated and mechanism-based application of training interventions to prevent specific injuries in different sports [11]. Specifically, it is unclear whether the timing and magnitude of underlying adaptations are noticeably different in regimes with higher training frequencies but low volume. Further, the potential benefits in high-risk populations, particularly female team sport athletes, are yet to be fully understood. Such knowledge would help to develop customized injury prevention programs in professional and recreational sports.

Thus, our aim was to investigate the effects of a neuromuscular warm-up intervention targeting lower extremity injury prevention on static and dynamic balance in competitive female team sport athletes. A secondary aim was to explore the time course of adaptations to the warm-up program. For this, balance was examined continuously at five different time points throughout the course of the neuromuscular training phase. Based on previous studies, it is hypothesized that all balance measures will improve with increasing time but may respond differently to progression of neuromuscular training.

## Methods

### Participants

Fourty-one female handball players (age: 24.0 ± 5.9 years; height: 170.2 ± 6.0 cm; weight: 65.7 ± 7.2 kg) were randomly allocated into an intervention and a control group using a simple coin toss. Athletes were recruited from three local handball teams (4^th^ division). Exclusion criteria were any lower extremity injury (acute or overuse) that prevented the player from regular participation in competition or practice at the time of the study. Assessments were performed before team practice sessions. All athletes were given detailed information on the study and written informed consent was obtained. The study was approved by the local ethics committee (FAU Erlangen-Nürnberg, Ref.-No. 155_15Bc) and conducted in accordance with the Declaration of Helsinki. The trial was retrospectively registered (trial registration number NCT02925377).

### Neuromuscular training program

The neuromuscular training program was implemented in each of the three participating handball teams. One week before the start of the neuromuscular training period athletes were informed which group they were assigned to. All of them were instructed to follow their usual physical activity routine and not to start new exercises or training programs outside their regular handball practice during the study period. The intervention group replaced their regular warm-up routine with neuromuscular warm-up exercises performed for 15 min prior to each practice session (3x/week) over a period of eleven weeks. The exercise components were based on a soccer-specific warm-up program [7, 22, 23] which was adapted to the handball setting. Accordingly, the neuromuscular training protocol included running, agility, balance, strength and plyometric components (see supplementary data).

The athletes in the intervention group started with two to three minutes of straight-ahead running at a low intensity. This was followed by two to three sets of each of the strength, balance and plyometric exercises, performed for 20–30 s. The following exercises were performed: planks and side planks (with hip or leg raise), single-leg stance (with increasing challenge), squats with toe rise (double-leg and single-leg) or walking lunges, jumping (vertical or lateral), bounding (running with large steps and high knee lifts), and running with directional changes (plant and cut). The exercises were performed with a special emphasis on movement quality. Each of the individual exercises had different levels of difficulty, which were individually progressed after three and six weeks. Increased challenge was realized by reducing the base of support, dual task conditions (e.g. ball catching and throwing during single-leg stance), or increasing exercise intensity (e.g. single leg squats) and duration. The intervention group was introduced to the neuromuscular warm-up program in the first week and familiarized with the proper technique of each exercise by verbal feedback. Additionally, a manual with detailed descriptions of the individual exercises and was provided. In the following, the athletes trained independently without direct supervision or feedback by the team’s coach. Adherence was recorded using exercise diaries. After finishing the neuromuscular warm-up protocol, the intervention group continued with regular handball practice.

Athletes in the control group followed their regular 15 min warm-up routine provided by the team coaches. The content varied across the teams, but typically included repeated running exercises at moderate intensity, followed by handball-unspecific warm-up exercises (without balancing, jumping or strengthening components). Due to organisational constraints, both the intervention and control groups in each team performed the warm-up exercises in the same location. The subsequent regular handball-specific training was performed with both groups together.

### Balance assessments

Static and dynamic balance tests were performed at baseline (T0), after three (T1), six (T2), and nine (T3) weeks, as well as post-intervention at week eleven (T4). An additional measurement was performed one week before baseline testing in order to familiarize participants to the testing protocol. The variables used for data analysis were: 1) dynamic: Maximum reach distances in the star excursion balance test (SEBT), and 2) static: Center of pressure (COP) sway velocity during single-leg quiet standing. All tests were performed barefooted on the athlete’s take-off leg and conducted in a fixed order by the same assessors. The assessors were not blinded to group assignment.

Dynamic balance was assessed using the SEBT. We performed four of the original eight directions: anterior, medial, lateral, posterior [24]. The athlete stood with the hands on the hips on the take-off leg in the center of a grid placed on the floor with four lines extending in anterior, posterior, medial and lateral direction. The athletes were instructed to reach the free leg as far as possible in each of the four directions, touch lightly on the line, and return to a double-leg stance in the center. Each individual completed one familiarization trial in each direction before the actual measurement. Two trials were recorded for each direction and the examiner manually recorded the maximum distance (cm). If athletes lost balance and touched the floor with the free limb before reaching start position or lifted hands off their iliac crests, the trial was discarded and repeated.

Postural sway, as a measure of static balance, was assessed on a Kistler force plate (model 9260AA6; Kistler Instrumente GmbH, Germany). The athlete had to stand on the take-off leg quietly for 20 s with eyes open, hands in hips, and the free foot touching the standing leg at the medial side of the shank. Failed attempts (non-balancing foot touched the floor or hands off hips, lifting hands off iliac crests, stepping, stumbling, falling, lifting the forefoot or heel) were discarded and repeated.

### Data analysis

The SEBT maximum reach distances in anterior, medial, lateral and posterior directions were normalized for individual leg length: normalized reach distance = reach distance (cm) * 100/leg length (cm). The average of the two measurements was used for data analysis.

Force plate data were used to calculate COP velocity (vCOP). Data were sampled at 180 Hz (Bioware, Kistler Instruments AG, version 4.0.1.2) and low-pass filtered at a cut-off frequency of 14 Hz using a second-order Butterworth filter [24]. vCOP (cm/s) was defined as the sum of the cumulated mean of medial-lateral and anterior-posterior COP displacement divided by the total time. The average of the two measurements was used for data analysis.

### Statistics

Linear mixed models were specified for each of the dependent variables. Maximum SEBT reach distances and COP sway velocity were defined as dependent variables and analyzed separately in linear regression models. The experimental factors ‘group’ and ‘time’ (continuously) as well as two-way interactions (group*time) were included as fixed factors. Athletes were included as a random factor and a random intercept model was computed to allow for individual variability. Restricted maximum likelihood was used to estimate variance components. Baseline differences between groups in demographic and balance measures were analysed using student’s t-tests. In an exploratory analysis, paired t-tests were used to investigate univariate level changes between baseline and the different time points within each treatment group. All statistical analyses were performed with SPSS statistics (IBM, version 22.0) software.

## Results

### Participant characteristics

21 participants were randomized to the intervention group and 20 to the control group. Table 1 demonstrates the participant’s characteristics. At baseline, there were no statistically significant differences between groups in age, mass, height, and body mass index.

**Table 1:** Participant's demographics (mean, SD)

	Controls		Intervention group		Group difference
	(*n* = 20)		(*n* = 21)		
	mean	SD	mean	SD	*P* value
Age (years)	24.00	5.59	23.95	6.27	0.98
Mass (kg)	65.90	6.77	65.52	7.69	0.87
Height (cm)	168.95	4.57	171.43	7.02	0.19
BMI (kg/m^2^)	23.08	2.13	22.28	2.05	0.23
SEBT (% leg length)	95.75	6.68	96.64	9.50	0.74
posterior	106.80	8.49	107.18	9.30	0.90
anterior	92.26	7.12	91.49	10.87	0.79
medial	100.96	7.74	101.29	11.37	0.92
lateral	82.99	8.95	86.60	11.17	0.27
vCOP (cm/s)	3.13	0.45	3.76	1.03	0.02

*BMI* Body mass index, *SEBT* star excursion balance test, *vCOP* center of pressure sway velocity

### Compliance

**Fig. 1:**
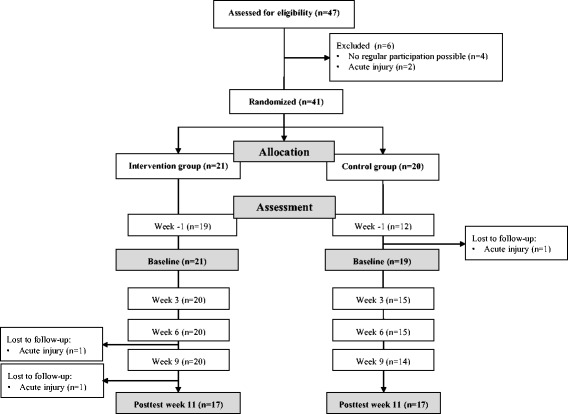
Participant´s flow

During the intervention period, players in the intervention group participated in 88.7 % (±12.1 %) of the 33 neuromuscular warm-up sessions. Two players in the intervention group and one player in the control group dropped out during the study period due to acute injuries unrelated to the intervention. Figure 1 demonstrates the participant’s flow. Not all participants were able to complete all assessments. Reasons for missed test sessions were illness or occupational duties.

### Balance outcomes

**Fig. 2:**
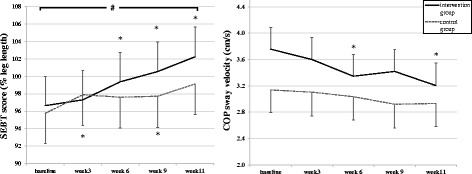
Time course of changes in the SEBT score and COP sway velocity in the intervention group (*bold line*) and controls (*dotted line*). Error bars indicate 95 % confidence intervals. * indicates significantly different from baseline (*p* ≤ .05). # indicates significant group * time interaction effect (*p* ≤ .05)

At baseline, COP sway velocity (*p* = 0.02) differed significantly between groups. Figure 2 illustrates the time course of the different balance measures. SEBT and vCOP responded differently to the neuromuscular intervention (Table 2).

**Table 2:** Statistical (fixed) effects (*p* - values) for time and group by time interactions

		Improvement pre-post intervention		
		Intervention group	Control group	Interaction
SEBT	mean	<0,001	0.002	0.014
	anterior	<0,001	0.002	0.253
	medial	<0,001	0.130	0.049
	lateral	<0,001	0.001	0.047
	posterior	<0,001	0.185	0.030
vCOP		<0,001	0.056	0.098

In the intervention group, measures for all SEBT reach directions improved over time. In the control group, this was the case for anterior and lateral direction but not for medial and posterior direction (Table 2). A significant group by time interaction was found for the SEBT mean score (*p* = .014), indicating greater improvements in the intervention group compared to the control group. The largest improvements existed from baseline to week 11 with an average improvement in SEBT score of 5.48 (±5.3) in the intervention group, compared to 3.45 (±4.6) in the control group. Paired t-tests revealed that the first significant improvements in the intervention group (*p* < .001) were observed after 6 weeks of training (Table 3). Linear trends differed significantly between groups for all individual SEBT reach directions (*p* < .05) except for anterior SEBT, with the largest improvements observed in lateral direction (intervention: +7.50; control: +5.92).

**Table 3:** Changes (mean, SD) between baseline (week 0) and weeks 3, 6, 9, and 11

Variable	Intervention Group								Control Group							
	0–3 weeks		0–6 weeks		0–9 weeks		0–11 weeks		0–3 weeks		0–6 weeks		0–9 weeks		0–11 weeks	
	*n* = 19		*n* = 20		*n* = 20		*n* = 17		*n* = 14		*n* = 14		*n* = 14		*n* = 17	
	mean	SD	mean	SD	mean	SD	mean	SD	mean	SD	mean	SD	mean	SD	mean	SD
SEBT score^a^	0.65	3.30	2.78*	2.76	3.85*	3.83	5.48*	5.33	2.32*	4.12	1.99	3.29	1.71*	4.83	3.45*	4.63
anterior	0.17	3.49	2.99*	3.58	2.77*	4.47	4.17*	5.18	1.20	4.53	0.46	3.20	1.32	4.41	3.73*	4.65
medial	0.90	3.61	1.88*	3.97	3.52*	5.45	4.26*	7.02	0.93	4.01	0.49	3.58	1.42	6.25	2.06	7.23
lateral	0.70	7.17	3.45*	5.74	5.63*	6.57	7.50*	8.30	5.59*	5.17	5.08*	6.00	3.19	8.22	5.92*	6.75
posterior	0.83	3.79	2.80*	4.10	3.48*	5.23	5.98*	5.70	1.57	7.97	1.91	6.26	0.92	6.41	2.10	6.81
vCOP (cm/s)	−0.15	0.73	−0.42*	0.65	−0.32	0.76	−0.61*	0.66	−0.14	0.32	−0.07	0.58	−0.21	0.57	−0.22	0.48

^a^SEBT scores normalized to leg length; *indicates significantly different from baseline (*p* ≤ .05)

Mean COP sway velocity improved significantly over time in the intervention group (−14.4 %; *p* < .001), and a trend for improvements existed in the control group (−6.2 %; p = 0.056). Tested effects did not differ significantly between groups (*p* = .098). Paired t-tests revealed that the first significant improvement in the intervention group (*p* < .001) was observed after 6 weeks of training (Table 3).

## Discussion

Our primary aim was to investigate the effects of a high frequency (3x per week), low volume (15 min) neuromuscular warm-up program on intrinsic injury risk factors in female team handball players. The results confirmed that eleven weeks of neuromuscular warm-up incorporated in the regular team routine was effective in improving dynamic, but not static balance in this population. Additionally, as a unique feature of this trial, we obtained balance outcomes repeatedly throughout the intervention. In agreement with previous trials [13, 16, 21], the time course of adaptations revealed that training effects appeared to occur not before 6 weeks of training and further progressed in the following.

The results of this study suggest that short bouts of neuromuscular training incorporated into regular warm-up routine can positively influence intrinsic injury risk factors in female handball players. In accordance with our findings, Holm et al. [13] reported dynamic balance improvements after eight weeks of low-volume neuromuscular training in female handball players. Exercises were performed three times a week for 15 min. Two other studies [15, 16] reported improvements of dynamic balance after six and eight weeks of neuromuscular training in female team sport athletes, however, training volume was much higher in these trials. Daneshjoo et al. [14] and Zech et al. [21] reported improved dynamic balance with short-term (10–12 weeks) neuromuscular warm-up programs in male team sport athletes. Taken together, the above-described findings suggest that the incorporation of neuromuscular training components regularly into the team warm-up routine may be a promising strategy to positively influence injury risk factors in ball sport athletes. Further, they provide a potential explanation for the success of neuromuscular warm-up programs in reducing injury rates in various team sport [9–12]. They would consequently result from improvements in neuromuscular control, leading to enhanced dynamic balance and movement coordination, which in turn would decrease the risk of harmful joint loading.

A unique feature of this trial was the repeated assessment of balance throughout the intervention phase in order to explore the time course of adaptations. Based on our exploratory analysis of the data, we found that the first significant balance improvements in the intervention group appeared to occur after six weeks of neuromuscular training and continued to improve until week 11. This is supported by findings from McLeod et al. [16] showing balance improvements after six weeks of neuromuscular training in female athletes. It is interesting to note, that the training had a much higher volume, but was performed less frequently (90 min, 2x/week). Our results indicate that lower exercise durations but higher weekly training frequencies might be sufficient to obtain meaningful balance improvements. This is further supported by a recent study demonstrating improvements after ten weeks of a neuromuscular warm-up training in male athletes [21]. Consequently, the overall training volume seems to be a key factor for adaptations in balance control and short but frequent training bouts a promising strategy. Thus, the incorporation of neuromuscular training components into the regular warm-up routine seems to be a practicable and efficient approach for injury prevention.

We investigated time-dependent changes in both, static and dynamic balance in this trial. Our results indicate that dynamic, but not static balance adapted to the neuromuscular warm-up program. Our results are in line with findings from two recent trials showing enhanced dynamic, but not static balance with a neuromuscular warm-up [21, 25]. This fact adds to a growing body of evidence suggesting that static and dynamic balance may respond differently to exercise interventions [13, 21, 25]. The neuromuscular warm-up program was developed on the basis of effective injury prevention interventions described in previous studies [7, 8, 11, 23]. Hence, the use of multiple exercise components (i.a. strength, plyometrics, flexibility) may not have been adequate to cause improvements in dynamic but not static balance. It is important to note that SEBT and postural sway also showed an improvement over time in the control group. We found a similar trend in a comparable trial [21]. Thus, it seems plausible that the repetitive measurement of balance variables may have introduced a test learning effect, which needs to be considered when interpreting the improvements of the intervention group.

The limitations of this study include the lack of assessor blinding, as well as blinding of the participants. Further, the intervention and control groups of each team performed their warm-up programs in the same gym, which may have led to a contamination effect. Additionally, improvements over time in the control group indicate a potential test learning effect, introduced by the repeated test sessions. This could have blurred the intervention effects to some extent. Lastly, it was not possible to collect data for each participant at all time points, which lowers the statistical power of our analyses.

## Conclusions

Eleven weeks of neuromuscular warm-up had beneficial effects on selected balance measures in female team handball players, indicating that this type of intervention can successfully modify an important injury risk factor. The course of adaptations suggests that a training volume of three times weekly over six weeks is effective to produce measurable improvements. Our findings suggest that neuromuscular training components improve dynamic rather than static balance. Further, in this specific population, the SEBT appears to be a sensitive measure to detect training related improvements in injury risk factors. It remains speculative whether a long-term use of the intervention will further increase the training benefits. In consideration of the specificity of the population and dose of intervention, more studies with athletes at an increased injury risk are needed to allow individualized neuromuscular training interventions to improve intrinsic injury risk factors.

## Additional files

Additional file 1: Master data. The file includes a table with the full dataset of this study. (XLSX 22 kb)
Additional file 2: Training program. The file includes a detailed description of the neuromuscular warm-up program implemented in this study. (DOCX 17 kb)

## Abbreviations

ACL: Anterior cruciate ligament
BMI: Body mass index
COP: Center of pressure
SEBT: Star excursion balance test
vCOP: Center of pressure sway velocity.
