# Supplementary material for: Time-dependent postural control adaptations following a neuromuscular warm-up in female handball players: a randomized controlled trial
Source: BMC Sports Sci Med Rehabil. 2016 Oct 13;8:33. doi: 10.1186/s13102-016-0058-5 (PMC5064777; doi:10.1186/s13102-016-0058-5)
Supplement: Additional file 2: — Training program. The file includes a detailed description of the neuromuscular warm-up program implemented in this study. (DOCX 17 kb) [file 13102_2016_58_MOESM2_ESM.docx]

**Neuromuscular warm-up program**(adapted from Soligard et al., 2008)

**A - Running Exercises (low intensity) (2-3 min)**

Low intensity straight-ahead jogging.

**B - Neuromuscular Exercises (strength – plyometrics – balance) (10 min)**

| **Level 1** | **Level 2** | **Level 3** |
| --- | --- | --- |
| **1 - Planks (straight / alternate legs / onle leg lift)** | | |
| **Starting position:** Lie on your front, supporting yourself on your forearms and feet. Your elbows should be directly under your shoulders.  **Exercise**: Lift your body up, supported on your forearms, pull your stomach in, and hold the position for 20-30 sec. Your body should be in a straight line. Try not to sway or arch your back.  **20-30s.3 sets.** | **Starting position:** Lie on your front, supporting yourself on your forearms and feet. Your elbows should be directly under your shoulders.  **Exercise**: Lift your body up, supported on your forearms, and pull your stomach in. Lift each leg in turn, holding for a count of 2 sec. Continue for 40-60 sec. Your body should be in a straight line. Try not to sway or arch your back.  **40-60s.3 sets.** | **Starting position:** Lie on your front, supporting yourself on your forearms and feet. Your elbows should be directly under your shoulders.  **Exercise**: Lift your body up, supported on your forearms, and pull your stomach in. Lift one leg about 10-15 centimetres off the ground, and hold the position for 20-30 sec. Your body should be straight. Do not let your opposite hip dip down and do not sway or arch your lower back. Take a short break, change legs and repeat.  **20-30s. 3 sets.** |
| **2 - Side Planks (static / hip raise / with leg lift)** | | |
| **Starting position**: Lie on your side with the knee of your lowermost leg bent to 90 degrees. Support your upper body by resting on your forearm and knee. The elbow of your supporting arm should be directly under your shoulder.  **Exercise**: Lift your uppermost leg and hips until your shoulder, hip and knee are in a straight line. Hold the position for 20-30 sec. Take a short break, change sides and repeat.  **20 – 30s / side. 3 sets** | **Starting position**: Lie on your side with both legs straight. Lean on your forearm and the side of your foot so that your body is in a straight line from shoulder to foot. The elbow of your supporting arm should be directly beneath your shoulder.  **Exercise**: Lower your hip to the ground and raise it back up again. Repeat for 20-30 sec. Take a short break, change sides and repeat.  **20 – 30s / side. 3 sets** | **Starting position**: Lie on your side with both legs straight. Lean on your forearm and the side of your foot so that your body is in a straight line from shoulder to foot. The elbow of your supporting arm should be directly beneath your shoulder.  **Exercise**: Lift your uppermost leg up and slowly lower it down again. Repeat for 20- 30 sec. Take a short break, change sides and repeat.  **20 – 30s / side. 3 sets** |
| **3 - Single-leg stance (static / throw & catch / perturbation)** | | |
| **Starting position**: Stand on one leg.  **Exercise**: Balance on one leg whilst holding the ball with both hands. Keep your body weight on the ball of your foot. Remember: try not to let your knees buckle inwards. Hold for 30 sec. Change legs and repeat. The exercise can be made more difficult by passing the ball around your waist and/or under your other knee.  **30 sec / side. 2 sets.** | **Starting position**: Stand 2-3 m apart from your partner, with each of you standing on one leg.  **Exercise**: Keeping your balance, and with your stomach held in, throw the handball to one another. Keep your weight on the ball of your foot. Remember: keep your knee just slightly flexed and try not to let it buckle inwards.  **30 sec / side. 2 sets.** | **Starting position**: Stand on one leg opposite your partner and at arm’s’ length apart.  **Exercise**: Whilst you both try to keep your balance, each of you in turn tries to push the other off balance in different directions. Try to keep your weight on the ball of your foot and prevent your knee from buckling inwards.  **30 sec / side. 2 sets.** |
| **4 - Squats / Lunges (toe raise / walking lunges / one-leg)** | | |
| **Starting position**: Stand with your feet hip-width apart. Place your hands on your hips if you like.  **Exercise**: Imagine that you are about to sit down on a chair. Perform squats by bending your hips and knees to 90 degrees. Do not let your knees buckle inwards. Descend slowly then straighten up more quickly. When your legs are completely straight, stand up on your toes then slowly lower down again.  **30 sec. 2 sets.** | **Starting position**: Stand with your feet hip-width apart. Place your hands on your hips if you like.  **Exercise**: Lunge forward slowly at an even pace. As you lunge, bend your leading leg until your hip and knee are flexed to 90 degrees. Do not let your knee buckle inwards. Try to keep your upper body and hips steady. Lunge your way across the pitch (approx. 10 times on each leg) and then jog back.  **10x / leg. 2 sets.** | **Starting position**: Stand on one leg, loosely holding onto your partner.  **Exercise**: Slowly bend your knee as far as you can manage. Concentrate on preventing the knee from buckling inwards. Bend your knee slowly then straighten it slightly more quickly, keeping your hips and upper body in line.  Repeat the exercise 10 times on each leg. 2 sets.  **10x / leg. 2 sets.** |
| **5 - Jumping (vertical / lateral / box jumps)** | | |
| **Starting position**: Stand with your feet hip-width apart. Place your hands on your hips if you like.  **Exercise**: Imagine that you are about to sit down on a chair. Bend your legs slowly until your knees are fl exed to approx 90 degrees, and hold for 2 sec. Do not let your knees buckle inwards. From the squat position, jump up as high as you can. Land softly on the balls of your feet with your hips and knees slightly bent.  **30 sec. 2 sets.** | **Starting position**: Stand on one leg with your upper body bent slightly forwards from the waist, with knees and hips slightly bent.  **Exercise**: Jump approx. 1 m sideways from the supporting leg on to the free leg. Land gently on the ball of your foot. Bend your hips and knees slightly as you land and do not let your knee buckle inward. Maintain your balance with each jump.  **30 sec. 2 sets.** | **Starting position**: Stand with your feet hip-width apart. Imagine that there is a cross marked on the ground and you are standing in the middle of it.  **Exercise**: Alternate between jumping forwards and backwards, from side to side, and diagonally across the cross. Jump as quickly and explosively as possible. Your knees and hips should be slightly bent. Land softly on the balls of your feet. Do not let your knees buckle inwards.  **30 sec. 2 sets.** |

**C - Running Exercises (2-3 min)**

**Bounding**

Run with high bounding steps with a high knee lift, landing gently on the ball of your foot. Use an exaggerated arm swing for each step (opposite arm and leg). Try not to let your leading leg cross the midline of your body or let your knees buckle inwards. Repeat the exercise until you reach the other side of the pitch, then jog back to recover. **2 sets.**

**Running with Plant & Cut**

Jog 4-5 steps, then plant on the outside leg and cut to change direction. Accelerate and sprint 5-7 steps at high speed (80-90% maximum pace) before you decelerate and do a new plant & cut. Do not let your knee buckle inwards. Repeat the exercise until you reach the other side, then jog back. **2 sets.**

**Progression of Neuromuscular Exercises**

**Level 1 Week 1 – 3**

**Level 2 Week 4 – 6 if** - target exercise time was accomplished twice for exercise 1 & 2

- no instability or inaccuracy was evident in exercises 3 – 5

**Level 3** **Week 7 – 11** **if** - target exercise time was accomplished twice for exercise 1 & 2

- no instability or inaccuracy was evident in exercises 3 – 5

**Reference**

Soligard, T., Myklebust, G., Steffen, K., Holme, I., Silvers, H., Bizzini, M., Andersen, T. E. (2008). Comprehensive warm-up programme to prevent injuries in young female footballers: cluster randomised controlled trial. *BMJ (Clinical research ed.), 337,* a2469.
